# Swift thermal reaction norm evolution in a key marine phytoplankton species

**DOI:** 10.1111/eva.12362

**Published:** 2016-02-28

**Authors:** Luisa Listmann, Maxime LeRoch, Lothar Schlüter, Mridul K. Thomas, Thorsten B. H. Reusch

**Affiliations:** ^1^ Evolutionary Ecology of Marine FishesGEOMAR Helmholtz‐Centre for Ocean Research KielKielGermany; ^2^ Department of Aquatic Ecology, –EawagSwiss Federal Institute of Aquatic Science and TechnologyDübendorfSwitzerland

**Keywords:** adaptation, coccolithophore, experimental evolution, global warming, phytoplankton, reaction norm, temperature

## Abstract

Temperature has a profound effect on the species composition and physiology of marine phytoplankton, a polyphyletic group of microbes responsible for half of global primary production. Here, we ask whether and how thermal reaction norms in a key calcifying species, the coccolithophore *Emiliania huxleyi*, change as a result of 2.5 years of experimental evolution to a temperature ≈2°C below its upper thermal limit. Replicate experimental populations derived from a single genotype isolated from Norwegian coastal waters were grown at two temperatures for 2.5 years before assessing thermal responses at 6 temperatures ranging from 15 to 26°C, with pCO_2_ (400/1100/2200 μatm) as a fully factorial additional factor. The two selection temperatures (15°/26.3°C) led to a marked divergence of thermal reaction norms. Optimal growth temperatures were 0.7°C higher in experimental populations selected at 26.3°C than those selected at 15.0°C. An additional negative effect of high pCO_2_ on maximal growth rate (8% decrease relative to lowest level) was observed. Finally, the maximum persistence temperature (*T*_max_) differed by 1–3°C between experimental treatments, as a result of an interaction between pCO_2_ and the temperature selection. Taken together, we demonstrate that several attributes of thermal reaction norms in phytoplankton may change faster than the predicted progression of ocean warming.

## Introduction

Temperature has an overriding effect on species composition, photosynthetic performance and growth rates of marine phytoplankton (Eppley 1972; Raven and Geider 1988; Thomas et al. 2012; Boyd et al. 2013). The thermal physiology of phytoplankton species broadly corresponds to mean temperature values within their climate zone. For example, in tropical species, optimal temperatures and upper thermal limits are higher compared to temperate or polar species (Thomas et al. 2012, 2016; Boyd et al. 2013). There is also preliminary evidence that temperature niche width is correlated with the annual temperature variation, with temperate species displaying a wider temperature range than both tropical/subtropical and polar species (Boyd et al. 2013) although this pattern was not observed in a recent literature compilation (Thomas et al. 2016).

At the within‐species level, there are also appreciable differences in thermal responses on growth and photosynthesis rates (Brand 1982; Wood and Leatham 1992) that likely have a heritable basis (Zhang et al. 2014). In light of global change impacting all marine and terrestrial ecosystems today, such within‐population diversity may provide essential standing genetic variation for populations to track climate change via genotypic selection and hence adaptive evolution at the population level (Reusch and Boyd 2013; Collins et al. 2014).

Although there have been some recent evolution experiments in the phytoplankton to ocean acidification and warming (e.g., Lohbeck et al. 2012; Schlüter et al. 2014; Hutchins et al. 2015), we currently do not know the time scales over which thermal reaction norms evolve, the response in growth and other traits to a range of environmental temperatures. The question of how rapidly biologically meaningful differences in these reaction norms can arise via adaptive evolution is highly important to understanding future ocean biogeochemical cycles, as the environment for phytoplankton is changing on the timescale of decades (e.g., Boyd et al. 2013). As a tool relatively new to marine science, long‐term experiments can directly address whether and how organismal responses to global change may evolve at the population level (Collins et al. 2014; Sunday et al. 2014). Here, fast‐dividing marine microbes (approx. 1 cell division day^−1^) are prime examples for observing evolution in action over timescales of several months to years (Reusch and Boyd 2013; Collins et al. 2014; Hutchins et al. 2015).

The response of a given phytoplankton species or genotype to temperature is described by a thermal reaction norm, which is typically unimodal and left (negatively) skewed (Huey and Kingsolver 1989) as above the optimal temperature *T*_opt_, growth rates decline more rapidly than below it. The maximum of the curve depicting the optimal growth temperature is correlated with (and generally higher than) the mean environmental temperature at the locale from which a population or genotype has been isolated for laboratory cultivation, reflecting adaptation to local temperature conditions (Thomas et al. 2012, 2016). As in other ectothermic species (Huey and Kingsolver 1989), phytoplankton thermal reaction norms are evolutionarily constrained by thermal trade‐offs that prevent any one species from dominating across all temperatures found in the world's oceans (Boyd et al. 2013). For example, species can be categorized as having a narrow or a wide thermal niche (specialist vs. generalist), with maximal growth rates that are traded off against generalist growth performance and vice versa (Angilletta et al. 2003; Izem and Kingsolver 2005). Other conceivable trade‐offs could be envisaged between maximal persistence temperature, thus stress tolerance, and maximal growth rates, but experimental data from phytoplankton demonstrating such trade‐offs are lacking.

Our model species is the world's most abundant calcifying microalgae, the coccolithophore *Emiliania huxleyi* (Paasche 2002), that is one of the most intensely studied eukaryotic phytoplankton species with a near‐worldwide distribution. Recently, this species has also become a model for combining experimental evolution and phytoplankton ecology (Reusch and Boyd 2013). Selection experiments subjecting this important phytoplankton species have shown that rapid adaptation to ocean acidification is possible within the time frame of 1 year either through genotypic sorting or via the occurrence of novel mutations within asexually dividing replicate populations (Lohbeck et al. 2012). In terms of temperature adaptation, previous experiments have shown that this species can adapt to a temperature only 1–2°C below the maximal growth temperature by rapid adaptive evolution within a timeframe of 1 year (corresponding to ≈500 asexual generations). Interestingly, increases in fitness relative to control populations were amplified by simultaneous exposure to ocean acidification levels of 1100 and 2200 μatm (Schlüter et al. 2014). The work by Schlüter et al. (2014) only tested two temperatures in the assay experiment, a control temperature (15.0°C) and one a few degrees below the lethal threshold (26.3°C). It is thus currently unknown how the entire thermal reaction norm may have changed upon thermal adaptation in this species, as well as in any other phytoplankton species.

Here, we address whether or not selection for a single temperature close to the upper thermal limit (26.3°C) resulted in a reconfiguration of the entire reaction norm shape relative to populations evolving at 15°C, the approximate isolation temperature of the coccolithophore ecotypes at Bergen, Norway (Lohbeck et al. 2012). Note that all genotypes/replicate populations had been grown previously for 4 years at 15°C such that the warm temperature is a novel environment, while we do not deny that there is also some long‐term adaptation to 15°C still ongoing during the experimental phase of this study. This was studied in full factorial combination with two levels of ocean acidification (1100 and 2200 μatm pCO_2_) along with ambient controls (400 μatm pCO_2_). We were particularly interested if adaptation to high temperature also changed the optimal growth temperature *T*_opt_, maximum persistence temperature *T*_max_ (i.e., the temperature above which growth rate becomes negative), and maximal growth rates *μ*_max_ (Boyd et al. 2013; Thomas et al. 2016). Moreover, we studied possible trade‐offs, for example with respect to *T*_max_ *T*_opt_, and *μ*_max_. Evolution experiments are particularly suited to address trade‐offs because trait correlations, among the above three attributes of thermal reaction norms, have to evolve within an identical genetic background (Fry 2003).

## Material and methods

### Study species, culturing, and experimental design

The coccolithophore *Emiliania huxleyi* is the most abundant calcifying organisms in the world oceans, distributed from subpolar to subtropical waters (Paasche 2002). When forming blooms, their areal extent can be seen from outer space owing to the calcite platelets that reflect a proportion of the incoming solar radiation. Previous studies in *Emiliania huxleyi* have demonstrated swift evolutionary adaptation to ocean acidification and warming in asexual populations within the time frame of 1 year (approx. 500 asexual divisions) (Schlüter et al. 2014). Here, we built upon a previous CO_2_ (Lohbeck et al. 2012) and temperature selection experiment (Schlüter et al. 2014) and ask whether and how the entire thermal reaction norm differs in two sets of asexual experimental populations that evolved for 2.5 years under a control and one high temperature close to the upper thermal limit. Note that maximal water temperatures off Bergen, Norway, are at most 19°C (see August maxima at http://www.seatemperature.org/europe/norway/bergen-august.htm), thus while the control temperature is within the conditions encountered by the culture genotypes, this was not the case for the high temperature.

**Figure 1:**
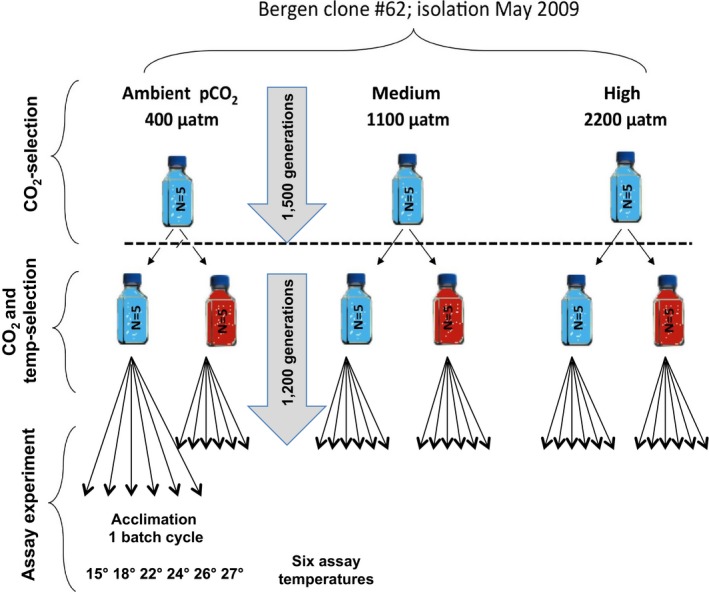
Five years of experimental evolution in *Emiliania huxleyi* in semi‐continuous batch cultures. Schematic representation of the experimental design and the selection history.

The temperature evolution experiment started in February 2013 when *Emiliania huxleyi* semi‐continual batch cultures at three CO_2_‐levels (*N *=* *5) were subdivided into a ‘cold’ (15.0°C) and a ‘warm’ (26.3°C) treatment, resulting in six fully factorial ‘temperature by CO_2_ treatment’ combinations (Fig. 1). Phenotypic changes after 1 year (approx. 500 asexual generations) of temperature selection, tested at only two assay conditions, that is, the two selection regimes, have already been published elsewhere (Schlüter et al. 2014). The exact level of the ‘high’ temperature was determined in pilot experiments because initially, daily specific growth rates were approximately similar at both temperatures, thus the elapsed number of generations would also be similar across any occurring evolutionary adaptation (Schlüter et al. 2014). The temperature treatment was run for 1200 asexual generations or 2.5 years, thus ≈700 generations longer than the results reported in Schlüter et al. (2014). The original selection lines were founded in 2009 from a single cell isolated from a natural phytoplankton assemblage in the coastal waters off Bergen (Lohbeck et al. 2012). *Emiliania huxleyi* cultures were uni‐algal but not axenic as checked by monthly light microscopy and flow cytometry.

Three CO_2_ levels were combined with the two temperatures in a full factorial way: along with a control treatment at 400 *μ*atm, we subjected *E. huxleyi* to 1100 μatm as simulation of an end‐of‐the‐century levels and 2200 μatm as the highest possible future level of ocean acidification in the year 2300 (Caldeira and Wickett 2003). CO_2_ levels were reached by bubbling the medium with CO_2_‐enriched air before inoculating the experimental populations at the respective temperature treatments for 24 h, see (Schlüter et al. 2014) for details on the seawater chemistry and CO_2_/carbonate measurements.

For long‐term experiments and temperature assays, we used artificial seawater ASW according to Kester et al. (1967) with the following nutrient additions: 64 μmol kg^−1^ of nitrate, 6 μmol kg^−1^ phosphate, trace metals, and vitamins according to f/8 composition, 10 nmol kg^−1^ of selenium, and 2 mL kg^−1^ filtered North Sea water to avoid limitation by micronutrients.

Cultures were kept under continuous rotation (0.5 min^−1^ in two Sanyo MLR‐351 light cabinets) at 150 μmol m^−2^ s^−1^ photon flux density under a 16:8 light:dark conditions during each 5‐day cycle. To start the next batch cycle, and renew the medium, every 5 days 10^5^ cells were transferred from cultures into fresh medium. The cell abundance and diameter were measured in triplicate before each transfer, using a Beckman Coulter Z2 Particle and Size Analyzer. Daily specific growth rates (*μ*) were calculated from cell abundance as *μ * =  (ln N_d_–ln N_0_)/d. All culture work, including the ASW preparation, was performed under sterile conditions (laminar flow) (Schlüter et al. 2014).

### Assay experiment to determine the thermal reaction norm

The reaction norm of all 30 replicates was determined at six assay temperatures after one full cultivation cycle of transfer of 100 000 cells to a new culture flask for acclimation (5 days =5‐8 cell divisions), namely at 15, 18, 22, 24, 26, and 27°C. While the two lower temperature values are attained at Bergen (Norway) coastal waters, the isolation location of the tested *E. huxleyi* genotype, we were particularly interested in the possible changes of upper range of temperature tolerance. This also implies that our design inappropriate to address correlated responses at low temperatures (i.e., <15°C). Batch cycles to assess growth rates differed in length because we needed to maintain approximately similar maximal cell numbers in the assays. To accommodate for the different growth rates, batch cycles lasted 6 days in the 15°C treatment, 5 days in the 18°C treatment, 4 days in the 22°C, 5 days for 24°C, 7 days (15°C) and 5 days (26.3°C) for 26°C, and 10 days (26.3°C) in the 27°C treatment. Preparation of ASW was similar to the long‐term culturing phase, except that during CO_2_ manipulation via bubbling the medium, the seawater was kept at the planned assay temperature (15°C, 18°C, 22°C, 24°C, 26°C, and 27°C (±3°C)) for 24 h, and not at the respective selection temperature (15.0 and 26.3°C). Nutrients were never limiting given the cell abundance achieved at the end of the batch cycles. At the end of each growth cycle, the cell densities and cell diameter were determined triplicate by a Z2 Coulter Particle Count and Size Analyzer (Beckman^®^ Coulter Counter; Krefeld, Germany), and specific daily growth rates (*μ*) were calculated from cell abundances as above.

### Statistical analyses

Where strains exhibited no growth at either 26 or 27°C, we removed the 27°C measurement from our calculations. The absence of growth can indicate either zero or negative growth rate, but we were unable to measure negative growth rates. Therefore, zero is almost certainly an overestimate at 27°C in these cases.

#### Thermal reaction norm parameter estimation

Thermal reaction norms are typically left‐skewed and have been described using a variety of functions. We used the equation and parameter and bootstrap‐based uncertainty estimation procedures described in Thomas et al. (2012) and Boyd et al. (2013).

(1) $$f\left(T\right)=ae^{bT}\left[1-{\left(\frac{T-z}{w/2}\right)}^{2}\right]$$

where specific growth rate *f* depends on temperature, *T*, as well as parameters *z*,* w*,* a,* and *b*. *w* is the temperature niche width (the range of temperatures over which growth rate is positive), while the other three (*z*,*a*,*b*) possess no explicit biological meaning but interact to influence the rate of increase in growth rate with temperature, the maximum growth rate and the optimum temperature for growth. We fit (1) to the growth data for each strain using maximum likelihood to obtain estimates for parameters *z*,* w*,* a,* and *b*. In addition, we estimated the optimum temperature for growth (*T*_opt_) and maximum persistence temperature (*T*_max_) through numerical estimation. For maximum growth rate *μ*_max_, we instead used the highest growth rate measured in our growth assays, as we are less confident in these estimates from our fitted reaction norms.

#### Estimating the influence of temperature selection and pCO_2_ levels on traits

We tested whether the three thermal traits changed as a result of selection at different temperatures and pCO_2_ levels while accounting for uncertainty in our estimates of these traits using a parametric bootstrapping approach. For each replicate, we fitted the thermal reaction norm function to the growth rate measurements and extracted the residuals from this fit. We then performed 1000 residual bootstraps, a procedure in which the residuals are randomly ‘reassigned’ to the predicted values (each of which corresponds to a growth rate measurement) and added to them, thereby generating a slightly different thermal reaction norm. For each iteration, we refitted the function and estimated the parameters (*z, w, a, b*) and also two of the derived traits, *T*_opt_ and *T*_max_.

Examining the distribution of the traits (*T*_opt_ and *T*_max_) over the 1000 bootstraps allowed us to quantify the uncertainty in our trait estimates, which we then incorporated in models seeking to explain how selection temperature and pCO_2_ influenced them. For each set of bootstraps of all 30 experimental units, we fitted a linear model explaining each trait as a function of selection temperature (coded as a categorical variable), pCO_2_ level and their interaction. pCO_2_ level was standardized by subtracting the mean and dividing by two standard deviations to improve fitting procedures and generate readily comparable model parameter values (Gelman 2008; Gelman and Su 2015. We then examined the distribution of linear model parameter estimates over the 1000 bootstraps to determine whether temperature selection, pCO_2_ or their interaction significantly influenced the traits. If the 95% confidence interval of a linear model parameter did not overlap zero, we concluded that the model parameter had a significant influence on the trait. If the interaction between selection temperature and pCO_2_ was an important predictor, we did not draw conclusions about significance of the main effects.

In the case of *μ*_max_, we fitted the linear model only the highest measured growth rate of each strain (no bootstraps), using selection temperature (coded as a categorical variable), pCO_2_ level and their interaction as explanatory variables. As with the other two traits, we standardized pCO_2_ level by subtracting the mean and dividing by two standard deviations. After this, we estimated the 95% confidence intervals on the fitted model parameters.

Parameter estimation and bootstrapping was performed in the R Statistical Environment 3.2.2. (R Core Team 2015).

## Results

**Figure 2:**
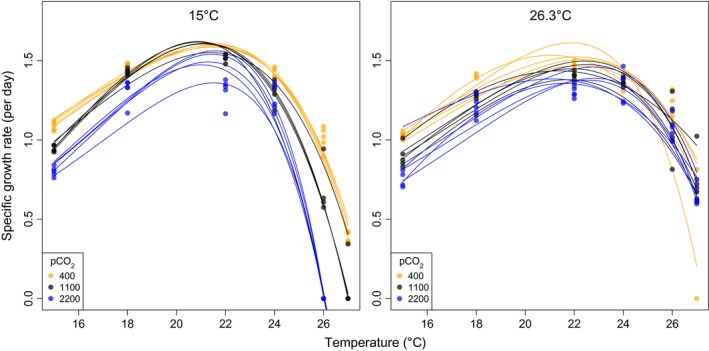
Fitted temperature reaction norms for individual replicate asexual populations according to eqn (1) in *Emiliania huxleyi*. Curves are grouped according to two different selection temperatures, 15.0 (left) and 26.3°C (right panel) fully crossed with three CO_2_ environments, depicted by color (orange = 400 μatm, black = 1100 μatm, blue = 2200 μatm). Note that the CO_2_ environment was similar during selection and temperature assay.

**Figure 3:**
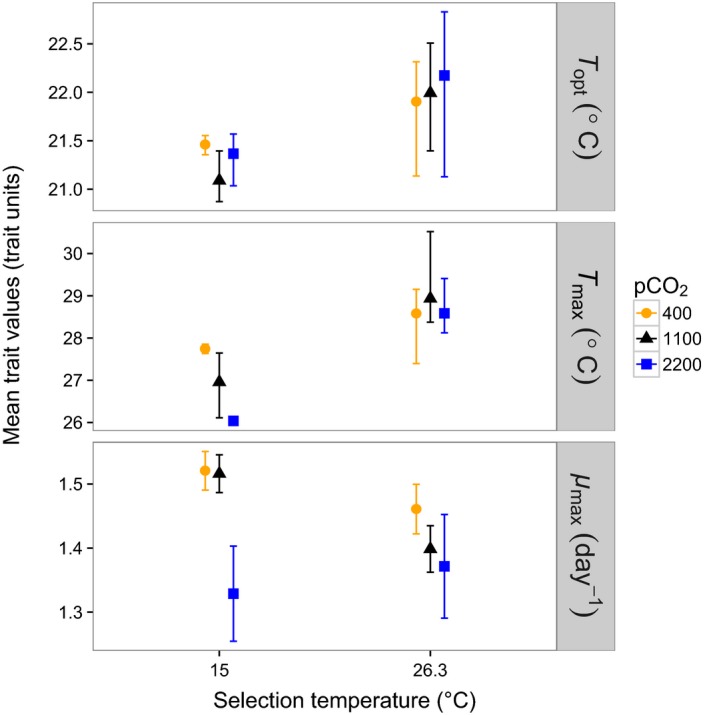
Mean temperature traits (*T*_opt_, *T*_max_, and *μ*_max_) in the different treatments. *T*_opt_ and *T*_max_ were estimated from reaction norm fits depicted in Fig. 2, while *μ*_max_ was calculated from the measured growth rates. Confidence intervals (±95%) are based on residual bootstraps of the fitted reaction norms for the first two traits; for *μ*_max_, calculations were based on the empirically measured growth rates and assumed normality of the data.

**Figure 4:**
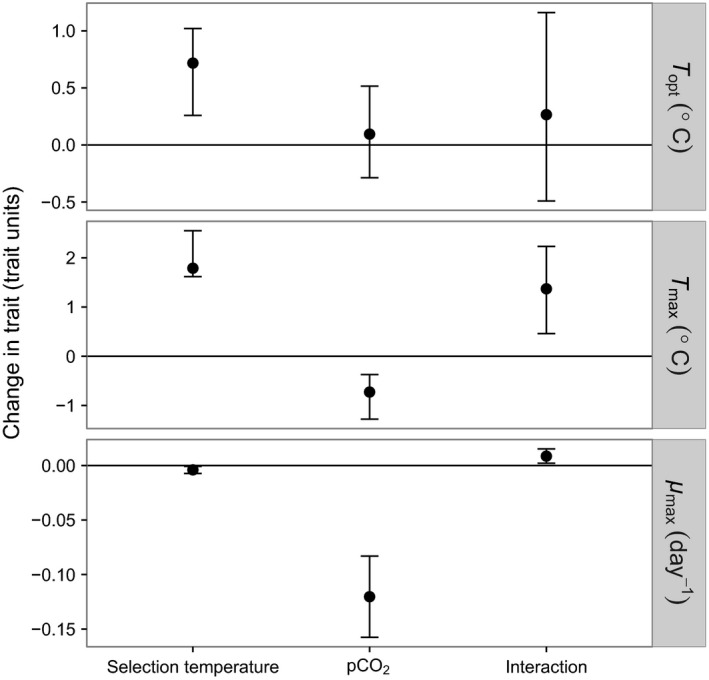
Changes in *Emiliania huxleyi* temperature traits *T*_opt_, *T*_max_ [based on the nonlinear curve fit according to eqn (1)] and *μ*_max_ (based on measured growth rates) as a function of selection temperature, pCO_2_, and their interaction. Standardized trait coefficients and their interaction are depicted; if confidence intervals do not overlap with zero, effects are statistically significant.

Fitted reaction norms of all five replicates within the two selection treatments are depicted in Fig. 2, grouped by selection treatment and CO_2_ condition. 2.5 years of selection at 15 and 26.3°C and three CO_2_ levels resulted in marked differences in reaction norm shape, which we capture in important temperature traits (Fig. 3). The effect sizes of the selection conditions (i.e., the regression coefficients of the parameters) on *T*_opt_, *T*_max_, and *μ*_max_ are presented in Fig. 4. The variance explained (adjusted *R* ^2^) by the models for these three traits was 0.37, 0.74, and 0.65, respectively. Temperature had a strong effect on *T*_opt_ and *T*_max_, while CO_2_ was important for determining the maximal growth rate G_max_ and for *T*_max_. Both *μ*_max_ and *T*_max_ were influenced by interactions between CO_2_ and selection temperature. We discuss the changes in the three traits below. We do not interpret our estimates of thermal niche width as we lack critical values at the lower temperature threshold to characterize them accurately.

### Optimum temperature for growth (*T*_opt_)

*T*_opt_ differed by 0.7°C on average between the low‐ and high‐temperature selection treatments (Fig. 3), being 21–21.5°C in the former and 22°C in the latter (Fig. 4). pCO_2_ did not have a detectable influence on *T*_opt_ either directly or as part of an interaction with temperature selection (Figs. 3 and 4).

### Maximum persistence temperature (*T*_max_)

*T*_max_ varied substantially between treatments and was influenced by an interaction between temperature selection and pCO_2_ level (Fig. 4). In the high‐temperature selection treatments, *T*_max_ reached 28.5–29°C regardless of pCO_2_ level (Fig. 3). However, in the low‐temperature selection treatments, *T*_max_ varied by 2°C depending on pCO_2_ level (Fig. 4). At 400 μatm (control), *T*_max_ was nearly 28°C, but this decreased by 1°C at 1100 μatm and 2°C at 2200 μatm (Fig. 3).

### Maximum growth rate (*μ*_max_)

*μ*_max_ was reduced strongly at higher pCO_2_ levels, but the extent of the reduction was influenced by an interaction with temperature (Figs. 3 and 4). *μ*_max_ decreased more strongly with increasing pCO_2_ at 15°C (by nearly 0.2 per day at 2200 μatm relative to 400 μatm) than at 26.3°C (by approximately 0.09 per day).

## Discussion

As a response to climate forcing via emission of greenhouse gasses, in particular CO_2_, atmospheric warming has already produced pronounced ocean warming during the past few decades in the world's oceans, and even down to depths of 2000 m (Roemmich et al. 2015). As a result, among diverse plankton species, observed range shifts have been attributed to mean ocean warming (or increased variability) and concomitant poleward shifts of distributional ranges (Poloczanska et al. 2013). Adaptive evolution is a nonmutually exclusive process to range shifts that allows populations to ‘stay’ within their geographical range and has recently come into the research focus (Hoffmann and Sgro 2011).

Here, we have addressed how the rate at which adaptive evolution will produce different thermal reaction norms in a genetically near‐uniform base population. We started our experimental evolution with a single isolate from Bergen, Norway. Thus, due to the lack of standing genetic variation, evolutionary adaptation most likely occurred via novel mutations. Mutant genotypes with under positive selection displayed higher Darwinian fitness and rose to (near) fixation in the asexually dividing population. Our scenario thus simulates that sexual reproduction and recombination is rare or absent (Reusch and Boyd 2013), which may actually be the case in some *E. huxleyi* populations (von Dassow et al. 2015). For our isolates, population‐level data indicate that they are composed of many genotypes (unpublished data), which mirror results from the English channel that even in bloom situations genotypes are in Hardy–Weinberg equilibrium (Krueger‐Hadfield et al. 2014) suggesting frequent sexual reproduction.

Although we started with one single genotype, we nevertheless found marked divergence among the two temperature selection treatments in several reaction norm parameters such as *T*_opt_, *T*_max_, and *μ*_max_. These effects of temperature selection interacted with the CO_2_ environment. Specifically, effects on *T*_max_, in particular, were markedly negatively affected by medium and high ocean acidification levels but only in high‐temperature selection treatments. This highlights how important interactions of major projected ocean perturbations are not only to understand future physiology, but also the evolutionary trajectories in plankton populations (Collins 2011; Schlüter et al. 2014). Note that our design cannot strictly test for temporal changes in reaction norms, because we cannot retest our initial base population or genotype as is possible in model systems such as *E.coli* or yeast with the help of cryopreservation (Elena and Lenski 2003). The salient experimental test is therefore the synchronic test of different evolution treatments at the same time, which also provides the necessary control for laboratory adaptation.

We cannot exclude that the genotype #62, originally isolated in 2009 off Bergen, Norwegian coastal waters, is special in terms of adaptation rates or magnitudes toward ocean warming and acidification. In other words, it is highly desirable to compare different genetic backgrounds with respect to their rates and magnitudes of adaptation, including associated phenotypic traits.

Our data support results of a shorter (1 year = 500 generations) evolution experiment reported in Schlüter et al. (2014). He tested thermal adaptation only at the two selection temperatures, 15.0°C and 26.3° over 1 year and found pronounced adaptation of growth rates to the long‐term selection temperature in reciprocal exposure assays. The relative fitness of high‐temperature‐adapted replicates compared to low‐temperature‐adapted ones (as quotient of exponential growth rates) was 1.08–1.25, when tested at 26.3°C. The higher the CO_2_ level in the experiment of Schlüter et al. (2014), the greater were the fitness gains of adapted populations, relative to the control populations evolving at today's pCO_2_ and at a temperature of 15°C. When focusing on the rather similar assay temperature 26°C assessed here, the present data reveal an even stronger thermal adaptation after about 700 additional generations of evolution. Depending on CO_2_, we find a relative fitness of 1.22 and 2.05 in ambient and medium (1100 μatm) pCO_2_. Note that at the highest CO_2_ level (2200 μatm), no growth was observed in the 15°C adapted populations at an assay temperature of 26°C. We also found a pronounced correlated response at 15°C – low‐temperature‐adapted populations grew faster at 15°C than high‐temperature‐adapted ones (Fig. 2).

Contrary to Schlüter et al. (2014), at an assay temperature of 26°C, we now find zero growth in some 15°C adapted replicate populations, which may be due to further adaptive evolution toward low‐temperature specialization. However, this may have been influenced by differences in assay methods. In the present study, the initiation of the assay experiment (which started with a 1‐week acclimation phase in all cases) was abrupt (i.e., within one single day), while the assay temperature was reached at a rate of 1°C per day in the earlier experiment by Schlüter et al. (2014). The concomitant carry‐over effects may have negatively affected growth rates in the 2nd assay cycle where the growth rates presented in this paper were measured.

In the ideal case, we would have started the thermal evolution experiment with identical genetic material at the onset. However, replicate lines had already 3 years time (about 1500 generations) to accumulate slightly favorable and concomitant hitchhiking mutations (Lang et al. 2013) under long‐term CO_2_ selection before thermal selection had started. Thus, the starting genetic diversity was likely higher than a pure uni‐clonal inoculum derived immediately from a single cell directly before the thermal selection started. This may explain the relatively fast pace and extent of reaction norm evolution compared to previous assessments of adaptation to ocean acidification (Lohbeck et al. 2012). Note, however, that also the CO_2_‐ambient control lines (at 400 μatm) always evolving under ambient CO_2_ levels had also changed their thermal reaction norm relative to the 26.3°C selected replicates, thus previous high‐CO_2_ selection was apparently not prerequisite for adaptive responses.

In any case, the standing genetic variation in natural phytoplankton populations is much higher: ample standing genetic variation in coccolithophores has often been observed in genetic marker studies (Iglesias‐Rodriguez et al. 2006; Krueger‐Hadfield et al. 2014) or physiological assessments (Brand 1982; Wood and Leatham 1992; Kremp et al. 2012; Zhang et al. 2014), which provide abundant possibilities for selection to operate.

The major result of evolution at different temperatures was that the optimal growth temperature *T*_opt_ shifted upwards, which was expected. As growth rates increased only little during the 5 years of selection at 15°C (Schlüter and Reusch, unpublished data), we attribute most of the divergence in reaction norm shape to changes at high temperature. This may reflect a trade‐off with between low‐ and high‐temperature performance. A second major finding is that *T*_max_ shifted upwards very strongly relative to the low‐temperature‐adapted populations, indicating that adaptation to lethal temperatures is possible over monthly‐to‐yearly timescales even in very small populations (relative to natural populations). While this upward shift was present in all high‐temperature selection replicates, pCO_2_ had a negative effect on *T*_max_ in the low‐temperature selection treatments. Currently, we have no explanation for this interaction, but it clearly deserves further testing in this and other phytoplankton species. In contrast to these two traits, *μ*_max_ was only weakly affected by temperature selection but strongly decreased with increasing pCO_2_, especially at 15°C. Unfortunately, since we could not assess the niche width, our findings cannot be interpreted within a generalist–specialist trade‐off scenario (Angilletta et al. 2003; Izem and Kingsolver 2005), However, it suggests that a general ‘flattening’ and broadening of the reaction norm may have occurred, superimposed onto a right‐hand shift of the entire reaction norm curve.

Although our upper thermal selection temperature was very unrealistic with respect to the temperatures *E. huxleyi* may experience throughout the North Atlantic (maximal temperatures at Bergen, the isolation site 19°C in August), our results nevertheless provide a proof‐of‐principle of swift evolution of reaction norms and provide first insights into trade‐offs of important traits associated with phytoplankton temperature reaction norms. Biogeochemical models of future ocean productivity contain thermal sensitivities of major phytoplankton groups as key parameters (Taucher and Oschlies 2011). Our results show that these thermal traits are parameters that can change by evolution and may to some extent track the expected increases in sea surface temperatures. A big open question is whether in the ocean, thermally sensitive species will be replaced by taxa possessing higher optimal growth temperatures and upper tolerances (i.e., ecological compositional change), or whether *in situ* evolution of thermal reaction norms will occur as a nonexclusive additional response, favouring the persistence of existing species and communities (Collins and Gardner 2009).

## Data archiving statement

Primary data are available through the data repository Pangaea (http://doi.pangaea.de/10.1594/PANGAEA.856736 ).
